# SAF-A/hnRNP U binds polyphosphoinositides via a lysine rich polybasic motif located in the SAP domain

**DOI:** 10.17912/micropub.biology.000761

**Published:** 2023-03-24

**Authors:** Amanda J Edson, Rhîan G Jacobsen, Aurélia E Lewis

**Affiliations:** 1 Department of Biological Sciences, University of Bergen, Bergen, Vestland, Norway

## Abstract

Polyphosphoinositides (PPIn) play essential functions as lipid signalling molecules and many of their functions have been elucidated in the cytoplasm. However, PPIn are also intranuclear where they contribute to chromatin remodelling, transcription and mRNA splicing. Using quantitative interactomics, we have previously identified PPIn-interacting proteins with roles in RNA processing/splicing including the heterogeneous nuclear ribonucleoprotein U (hnRNPU/SAF-A). In this study, hnRNPU was validated as a direct PPIn-interacting protein via 2 regions located in the N and C termini. Furthermore, deletion of the polybasic motif region located at aa 9-24 in its DNA binding SAP domain prevented PPIn interaction. In conclusion, these results are consistent with hnRNPU harbouring a polybasic region with dual functions in DNA and PPIn interaction.

**Figure 1.**
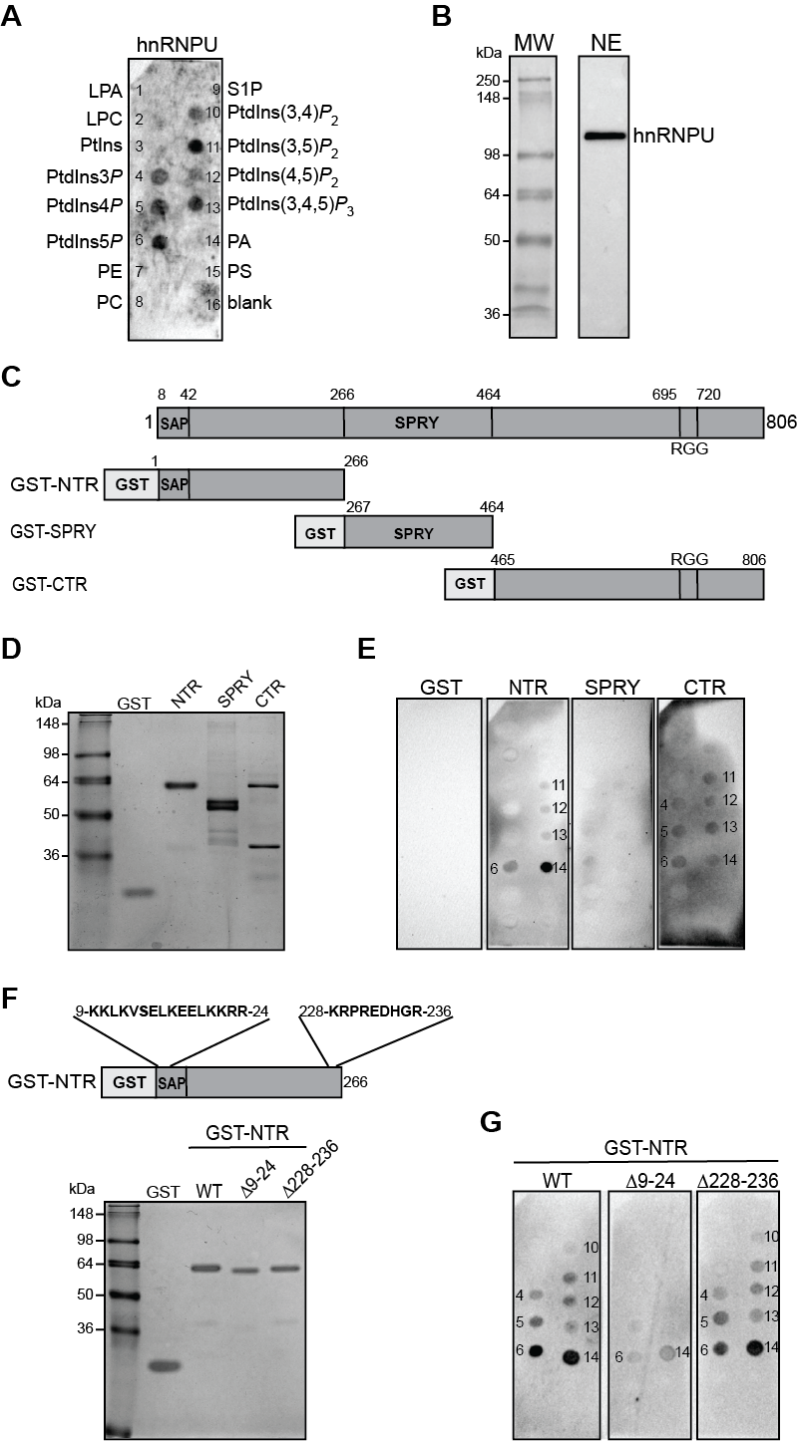
Interaction mapping of hnRNPU with polyphosphoinositides ( **A** ) Nuclei were isolated from actively growing HeLa cells and incubated with 5 mM neomycin. Dialysed neomycin-displaced supernatants (100 μg) were overlaid on PIP strips and protein-lipid interactions were detected with an anti-hnRNPU and anti-mouse IgG-HRP antibodies by chemiluminescence (n = 4). ( **B** ) Dialysed neomycin-displaced supernatants were resolved by SDS-PAGE and immunoblotted with anti-hnRNPU and anti-mouse IgG-HRP antibodies (n = 4). ( **C** ) Diagram showing the full length hnRNP-U detailing the location of the SAP (SAF-A/B, Acinus and PIAS), SPRY (Sp1A ryanodine receptor) domains as well as the RNA-binding RGG (arginine-glycine-glycine) box, according to uniprot ID Q00839 as well as the deletion constructs generated in this study fused to GST. ( **D** ) Purity analysis of GST and GST fused proteins with the N-terminal region (NTR), SPRY domain and C-terminal region (CTR) by SDS-PAGE and Coomassie blue staining. ( **E** ) Lipid overlay assay using PIP strips incubated with GST or GST-fused proteins. Protein-lipid interactions were detected using an anti-GST-HRP conjugated antibody with a longer exposure for GST, GST-NTR and -SPRY (450 sec exposure) and shorter exposure for GST-CTR (50 sec) due to higher background (n = 3 for NTD and SPRY, n = 2 for CTD). ( **F** ) Upper panel: Diagram showing the location of the polybasic regions in the NTR. Lower panel: Purity analysis of GST and GST fused proteins NTR WT and deletion mutations by SDS-PAGE and Coomassie staining. ( **G** ) Lipid overlay assay with the NTR without and with deletions of the indicated polybasic regions (n = 2). The numbers shown in E and G indicate the positions of spotted PPIns and PA as shown in Figure 1A.

## Description

Biomolecular interactions consisting of protein-lipid complexes are essential in cellular processes to allow appropriate biological responses (D’Santos and Lewis, 2012; Saliba *et al* ., 2015). Consequently, alteration of these interactions is implicated in many diseases (Wymann and Schneiter, 2008). This particularly applies to the signalling lipids, polyphosphoinositides (PPIn), which are derivatives of phosphatidylinositol (PtdIns), phosphorylated on three possible hydroxyl groups on the inositol headgroup. They can carry one (PtdIns3 *P* , PtdIns4 *P* and PtdIns5 *P* ), two (PtdIns(3,5) *P*_2_ , PtdIns(3,4) *P*_2_ , PtdIns(4,5) *P*_2_ ) or a maximum three (PtdIns(3,4,5) *P*_3_ ) negatively charged phosphate ((Choy *et al* ., 2017) and nomenclature based on Michell *et al* (Michell *et al* ., 2006)). PPIn are anchored in cellular membranes via their hydrophobic fatty acid tails while their differently phosphorylated headgroups act as signalling codes to recruit proteins harbouring PPIn-binding domains or polybasic amino acid clusters (Hammond and Balla, 2015). In addition, PPIn are present in the nucleus where they have been shown to play roles in chromatin remodelling, transcription, and mRNA processing via a few effector proteins (Fiume *et al* ., 2019; Hamann and Blind, 2018; Jacobsen *et al* ., 2019). To expand our understanding of how PPIn signals in the nucleus, we used an unbiased quantitative proteomics approach to enrich for and identify PPIn effector proteins (Lewis *et al* ., 2011). This approach was based on the incubation of isolated nuclei with the aminoglycoside neomycin, which binds to PPIn and hence displace PPIn effector proteins. Displaced proteins were identified and showed to have roles in mRNA processing, including heterogeneous ribonucleoproteins (hnRNP) U (alias Scaffold attachment factor A - SAF-A). hnRNPU was then identified in the PtdIns(4,5) *P*_2_ and PtdIns(3,4,5) *P*_3_ nuclear interactomes using this approach combined with PPIn pull downs (Lewis *et al* ., 2011; Mazloumi Gavgani *et al* ., 2021) or in the PtdIns4 *P* interactome using immunoprecipitation (Faberova *et al* ., 2020).

To first validate the interaction of hnRNPU with PPIn, we tried to express and purify GST-fused hnRNPU. The recombinant full length hnRNPU could not be expressed in bacteria and was unstable, consistent with a previous study (Kim and Nikodem, 1999). We therefore examined its PPIn binding properties by lipid overlay assay using neomycin-displaced protein supernatants obtained from HeLa nuclei followed by detection with an anti-hnRNPU antibody *(* ***Figure 1A*** *)* . We observed an interaction with all PPIn. The neomycin supernatants were also resolved by Western immunoblotting to demonstrate the specificity of the anti-hnRNPU antibody *(* ***Figure 1B*** *)* . To assess direct interaction, three deletion constructs were generated fused with GST and spanning the N-terminus region (NTR) which includes the SAP (SAF-A/B, Acinus and PIAS) domain, the SPRY (Sp1A ryanodine receptor) domain and the remaining C-terminal region (CTR) *(* ***Figure 1C*** *)* . GST and the GST fusion proteins were expressed and purified *(* ***Figure 1D*** *)* and used in lipid overlay assays *(* ***Figure 1E*** *)* . GST showed no signal while the NTR and CTR constructs showed interaction with PPIn, albeit with different signal intensity, the most intense signals detected being those with the CTR *(* ***Figure 1E*** *).* hnRNPU harbours two polybasic regions (PBR) in the NTR, one located within the SAP domain (9-KKLKVSELKEELKKRR-24) and the other located at aa 228-KRPREDHGR-236 *(* ***Figure 1F*** *)* . Considering the previously reported importance of such motif for PPIn interaction in nuclear proteins ((Karlsson *et al* ., 2016; Mazloumi Gavgani *et al* ., 2021; Viiri *et al* ., 2009) and reviewed in (Jacobsen *et al* ., 2019)), each PBR was deleted within the NTR. The WT and deletion mutants were expressed and purified *(* ***Figure 1F*** *)* and the effect of these deletions on PPIn binding was examined by lipid overlay assay *(* ***Figure 1G*** *)* . The first PBR (aa 9-24) was found to be required for PPIn binding *(* ***Figure 1G*** *)* , indicating specificity for this polybasic cluster. The SAP domain is known to bind to A-T rich areas of DNA (Gohring *et al* ., 1997; Kipp *et al* ., 2000) and these results suggest that the PBR located within the SAP domain may have dual functions in DNA binding and PPIn interaction. This has indeed been reported previously for the co-repressor sin3A-associated protein 30-like (SAP30L), where a PBR was shown to contribute to both DNA and PPIn interaction (Viiri *et al* ., 2009). In addition, the CTR may act as a second site for PPIn interaction.

## Methods

**Plasmids**

The NTR (aa 1-266), SPRY (aa 267-464) and CTR (aa 465-806) hnRNPU fragments were amplified by PCR from the pET-21a-hnRNPU construct (human, isoform 2 Uniprot Q00836, aa 1-806) obtained from Paul R Clarke (University of Dundee, UK (Berglund and Clarke, 2009)) using primers flanked by EcoRI/SalI restriction sites and cloned into pGEX-4T1 (Table 1). The pGEX-4T1-NTRD9-24 and -NTRD228-236 deletion mutants were generated by overlap extension PCR site directed mutagenesis. All plasmids were verified by sequencing using the ABI Prism BigDye Terminator version 3.1 cycle sequencing kit (Applied Biosystems).

**Protein expression and purification**

pGEX-4T1 containing the NTR, SPRY or CTR fragments of hnRNPU were transformed into *E. coli* BL21-RIL DE3. Bacterial cultures were grown at 37°C and further induced with 0.5 mM isopropyl-β-D-thiogalactopyranoside for 3 h at 37°C (NTR), or overnight at 26°C (SPRY) or 18°C (CTR). Bacterial cultures were centrifuged at 6000 g for 10 min at 4°C and pellets were resuspended in 0.1 M sodium phosphate pH 7.0, 1 mM DTT, 0.5 mg/ml lysozyme and 1x Sigma protease inhibitor cocktail (including 0.5% Triton-X100 for the CTR), incubated on ice for 30 min and sonicated 3 times for 30 sec at 4°C. NaCl was added to a final concentration of 0.2 M and lysates were further incubated for 10 min on ice and finally centrifuged at 13 000 g at 4°C for 15 min. The CTR fragment was insoluble and was extracted from pellets according to Tassan *et al* (Tassan et al., 1995). Pellets were lysed in 0.1 M Tris-HCl pH 8.5, 6 M urea overnight at 4°C rotating. The lysate was then centrifuged at 10,000 g at 4°C for 15 min. The supernatant was recovered and diluted 1:10 in 50 mM KH _2_ PO _4_ pH 10.7, 1 mM EDTA, 50 mM NaCl and incubated at room temperature for 30 min. The pH was adjusted to 8.0 allowing the protein to renature. The renaturation process proceeded for an additional 30 min at room temperature. The insoluble material was centrifuged at 10,000 g at 4°C for 5 min and the supernatant was saved for glutathione purification. Protein purification was performed using glutathione-agarose 4B beads. The GST fused recombinant protein was then eluted by adding elution buffer to the beads (50 mM tris pH 7.6, 250 mM NaCl, 10 mM KCl, 10 mM MgCl _2_ , 2 mM DTT, 20 mM reduced glutathione) and left to incubate at room temperature for 30-60 min. All protein preparations were analysed by SDS-PAGE and Coomassie staining.

**Cell culture, nuclear isolation and neomycin extraction**

HeLa cells were cultured in Dulbecco’s modified Eagles’ medium with 10% foetal bovine serum and 1% penicillin/streptomycin at 37 ^0^ C in a 5% CO _2_ incubator to about 80-90% confluence. Crude nuclear fractionation was performed as previously reported (Karlsson et al., 2016). Nuclei were washed with retention buffer containing 20 mM Tris pH 7.5, 70 mM NaCl, 20 mM KCl, 5 mM MgCl _2_ , 3 mM CaCl _2_ and protease inhibitor cocktail. The nuclei were then incubated with freshly prepared 5 mM neomycin (Neomycin trisulfate salt, Sigma-Aldrich) in retention buffer, rotating for 30 min at RT. After centrifugation at 13000 rpm for 5 min, the supernatant containing the neomycin-displaced protein extract was collected. Neomycin supernatants were dialysed three times in 900 mL of cold lipid pulldown buffer containing 20 mM HEPES pH 7.5, 150 mM NaCl, 5 mM EDTA, 0.1 % Igepal using Slide-A-Lyser Mini dialysis units (Thermo Fisher Scientific) for 1 h at 4°C each time. The protein concentration was measured using the bicinchoninic acid protein assay (Thermo Fisher Scientific).

**Western immunoblotting**

Dialysed neomycin supernatants were resolved by SDS-PAGE and proteins were transferred to a nitrocellulose membrane. The membranes were blocked with 7% skimmed milk in PBS-T (137 mM, NaCl, 2.7 mM KCl, 10 mM Na _2_ HPO _4_ , 0.05% Tween 20) and incubated with anti-hnRNPU (Santa Cruz, sc-32315, 1:10,000) overnight at 4°C, followed by anti-mouse secondary antibody conjugated with horseradish peroxidase (HRP) at 1:10,000 dilution for 1 h. The membranes were washed 6 x 5 min with PBS-T following each antibody incubation. The immunoreactive bands were visualised with enhanced chemiluminescence using SuperSignal West Pico Chemiluminescent Substrate (Thermo Fisher Scientific) according to the manufacturer’s instructions and detected with a BioRad ChemiDocTM Xrs+.

**Lipid overlay assay**

Lipid overlay assay were performed using PIP Strips ^TM^ from Echelon according to Karlsson *et al* (Karlsson *et al* ., 2016) incubated with 0.5 µg/mL of recombinant GST or GST-tagged hnRNPU deletion constructs, or with 100 μg of dialysed neomycin extracts. Visualization was achieved with anti-GST-conjugated to HRP (abcam, ab3416, 1:30,000) for the recombinant proteins or anti-hnRNPU (Santa Cruz, sc-32315, 1:10,000) followed by anti-mouse-HRP (Thermo Fisher Scientific, 32430, 1:10,000) for the neomycin extract, and enhanced chemiluminescence.

## Reagents

**Primers**

**Table:** 

**Primers** **used in PCR cloning**	**Sequence 5’-3’**
hnRNPU_NTD_Fwd	GAAGAT GAATTC ATGAGTTCCTCGCCTGT
hnRNPU_NTD_Rev	ATGCGA GTCGAC TCAATACTTGTTCTCTTC
hnRNPU_SPRY_Fwd	ATTCCG GAATTC AGCAGAGCCAAATCTCC
hnRNPU_SPRY_Rev	TGCCAA GTCGAC TCACTTTTCCTTCTGACC
hnRNPU_CTD_Fwd	TGCCAA GAATTC CCATATTTTCCAATACC
hnRNPU_CTD_Rev	TTCCG GTCGAC TCAATAATATCCTTGG

**Plasmids**

**Table:** 

**Name**	**Description**
pGEX-4T1-hnRNPU-NTR	Wild type construct of the N-terminal region (corresponding to aa 1-266) cloned in EcoRI and SalI sites
pGEX-4T1-hnRNPU-SPRY	Wild type construct of the SPRY domain (corresponding to aa 267-464) cloned in EcoRI and SalI sites
pGEX-4T1-hnRNPU-CTR	Wild type construct of the C-terminal region (corresponding to aa 465-806) cloned in EcoRI and SalI sites
pGEX-4T1-hnRNPU-NTR-ΔD9-24	Deletion of the corresponding aa 9-24 within the NTR construct
pGEX-4T1-hnRNPU-NTR-Δ228-236	Deletion of the corresponding aa 228-236 within the NTR construct
